# Responses to Salt Stress in *Portulaca*: Insight into Its Tolerance Mechanisms

**DOI:** 10.3390/plants9121660

**Published:** 2020-11-27

**Authors:** Orsolya Borsai, Mohamad Al Hassan, Cornel Negrușier, M. Dolores Raigón, Monica Boscaiu, Radu E. Sestraș, Oscar Vicente

**Affiliations:** 1 Institute of Life Sciences, University of Agricultural Sciences and Veterinary Medicine Cluj-Napoca, Mănăștur St. 3-5, 400372 Cluj-Napoca, Romania; orsolya.borsai@usamvcluj.ro; 2 AgroTransilvania Cluster, Dezmir, Crișeni FN, 407039 Cluj-Napoca, Romania; 3 Wageningen UR Plant Breeding, Wageningen University and Research Centre, 6708 PB Wageningen, The Netherlands; mohamed.alhassan@wur.nl; 4 Department of Soil Sciences, University of Agricultural Sciences and Veterinary Medicine Cluj-Napoca, Mănăștur St. 3-5, 400372 Cluj-Napoca, Romania; cornel.negrusier@usamvcluj.ro; 5 Institute for the Conservation and Improvement of Valencian Agrodiversity (COMAV), Universitat Politècnica de València, Camino de Vera s/n, 46022 Valencia, Spain; mdraigon@qim.upv.es (M.D.R.); ovicente@upvnet.upv.es (O.V.); 6 Mediterranean Agroforestry Institute (IAM), Universitat Politècnica de València, Camino de Vera s/n, 46022 Valencia, Spain; 7 Faculty of Horticulture, University of Agricultural Sciences and Veterinary Medicine Cluj-Napoca, Mănăștur St. 3-5, 400372 Cluj-Napoca, Romania; rsestras@usamvcluj.ro

**Keywords:** abiotic stress, antioxidant activity, growth inhibition, ion homeostasis, proline, salt stress

## Abstract

Climate change and its detrimental effects on agricultural production, freshwater availability and biodiversity accentuated the need for more stress-tolerant varieties of crops. This requires unraveling the underlying pathways that convey tolerance to abiotic stress in wild relatives of food crops, industrial crops and ornamentals, whose tolerance was not eroded by crop cycles. In this work we try to demonstrate the feasibility of such strategy applying and investigating the effects of saline stress in different species and cultivars of *Portulaca*. We attempted to unravel the main mechanisms of stress tolerance in this genus and to identify genotypes with higher tolerance, a procedure that could be used as an early detection method for other ornamental and minor crops. To investigate these mechanisms, six-week-old seedlings were subjected to saline stress for 5 weeks with increasing salt concentrations (up to 400 mM NaCl). Several growth parameters and biochemical stress markers were determined in treated and control plants, such as photosynthetic pigments, monovalent ions (Na^+^, K^+^ and Cl^−^), different osmolytes (proline and soluble sugars), oxidative stress markers (malondialdehyde—a by-product of membrane lipid peroxidation—MDA) and non-enzymatic antioxidants (total phenolic compounds and total flavonoids). The applied salt stress inhibited plant growth, degraded photosynthetic pigments, increased concentrations of specific osmolytes in both leaves and roots, but did not induce significant oxidative stress, as demonstrated by only small fluctuations in MDA levels. All *Portulaca* genotypes analyzed were found to be Na^+^ and Cl^−^ includers, accumulating high amounts of these ions under saline stress conditions, but *P. grandiflora* proved to be more salt tolerant, showing only a small reduction under growth stress, an increased flower production and the lowest reduction in K^+^/Na^+^ rate in its leaves.

## 1. Introduction

Adverse environmental conditions or abiotic stresses such as drought, soil salinity, low or high temperatures share their detrimental effect on the water status of plants, causing a reduction in their photosynthetic capacity and, consequently, limiting their vegetative growth and reproductive success [1,2]. Among the abiotic stresses mentioned, drought and salinity constitute the greatest threat to global food security [3], due to the extent and frequency of their occurrence, as well as their effects on the production of the yields of all major crops [4,5]. With increasing demand for food sources due to the booming world population, the need to combat the effects of drought and soil salinity (reduced crop production) becomes imperative [6]. This is highlighted by the fact that more than 800 million hectares of arable land are currently affected by drought and salinity, and salinity in particular is projected to affect 30% of agricultural land in the next 25 years and about 50% by the end of this century [7]. This increase in the extent of soil salinization (estimated at 1 to 2% per year [8]) is being aggravated by climate change, urbanization and pollution [9].

Soil salinization and drought overlap in inducing osmotic imbalance in affected plants [10]; however, the toxic influx of ions or ionic toxicity is salinity-specific [11]. Increased salinity restricts vegetative growth due to reduced photosynthetic capacity, as affected plants reduce their gas exchange by closing their stomata to reduce water loss [12,13]. As a result, further accumulation of reactive oxygen species (ROS) would begin, disrupting cellular processes and inducing oxidative damage, particularly to the photosynthetic machinery [14,15]. Affected plants respond by activating a series of constitutively expressed defense mechanisms to evade and mitigate the effects of such stresses [16], until optimal conditions are restored or successful propagation of the genetic material is ensured. These include the accumulation of soluble solutes or “osmolytes” for osmotic adjustment [17], the activation and over-expression of ROS sequestering enzymes such as catalase, ascorbate peroxidase and superoxide dismutase [18], and ionic avoidance [19] and compartmentalization [20].

The adaptation and response of plants to adverse environmental conditions, in particular soil salinization, differs greatly among affected species. This ranges from improved growth under stress conditions in the case of obligate halophytes, to complete cessation of growth, and even death for susceptible glycophytes [21]. Almost 98% of terrestrial plant species are considered glycophyte, including all major crops [22], this is partly due to the erosion of the natural resistance inherent to abiotic stresses over millennia of unidirectional breeding for yield, taste and color [23]. A strategy that is recently gaining ground among the scientific community in an attempt to combat the anticipated spread of soil salinization and its underlying effects on global food security is the development of salt-tolerant crops [24]. However, unlike plant resistance to biotic stresses, which depends mostly on monogenic traits, genetically complex responses to abiotic stresses are multigenic and therefore more difficult to control, breed and manipulate [25].

Therefore, a better understanding of the underlying mechanisms that confer tolerance is considered necessary to facilitate the development of more tolerant crops [24], especially because these response strategies differ greatly among plant groups [26]. One area that has been little explored is research on the stress responses of wild relatives of crops, halophytes and minor, ornamental and industrial crops, which could serve as an underexploited source of genetic tools for deciphering the fine tuning of crop tolerance. This would identify targets for molecular improvement and the development of stress markers, which could accelerate ongoing programs towards more stress resistant crops.

Salt tolerance in halophytes is developed through an efficient and well-defined coordination of physiological and metabolic pathways [27] that enable them to complete their life cycles even under conditions of high salinity [28]. The adaptation capacity of halophytes to salt stress is based on several physiological adjustments that take place both at the cellular and molecular level, among them osmotic regulation and ionic homeostasis through extrusion and/or compartmentalization [14]. In previous reports, the inhibitory effects of excessive saline stress on several physiological and biochemical processes in plants were highlighted. Among the former, the function of the photosynthetic apparatus is the most likely to be affected due to stomatal closure and the consequent reduction in carbon dioxide absorption under stress conditions [14]. Therefore, the effects of stress on photosynthesis can be easily evaluated by monitoring changes at the level of photosynthetic pigments [28,29]. It has also been observed that, under stress conditions, halophytes accumulate different compatible solutes, such as sugars (sucrose), betaine (glycinebetaine), amino acids (proline) and sugar alcohols (sorbitol) depending on phylogeny and functional needs [30,31,32]. In addition, different stressful environmental conditions were reported to induce oxidative stress in plants due to increased “reactive oxygen species” (ROS). However, in both halophytes and glycophytes, ROS production and detoxification occur under optimal conditions, although the balance is greatly altered in stressful situations, particularly high salinity [33]. To that extent, malondialdehyde (MDA), a product of lipid peroxidation that is considered a reliable marker of oxidative stress in plants, was examined in parallel with studies on stress for ROS accumulation. An increase in MDA content in response to abiotic stresses in different plant species is reported in several publications [34]. Since different environmental stresses cause secondary oxidative stress in plants, a general adaptive reaction of plants is the activation of antioxidant compounds and enzymes. A complex group of phenolic compounds and especially the subgroup of flavonoids that include many secondary metabolites are also very important for these antioxidant responses [35].

The genus *Portulaca* comprises approximately 130 species known today, including annuals and perennials (which grow in the tropics and subtropics) and are generally herbaceous, with hairs (conspicuous or inconspicuous) developed at the axils of the succulent, cylindrical leaves [36]. *Portulaca* species have a wide range of distribution throughout the world, from sea level to 2600 m of altitude. *Portulaca oleracea* is often used as a vegetable, but many of its varieties, as well as other species of this genus, are widely known as ornamentals, much appreciated for their flowers of diverse color, type and size, and for their multiple uses in garden design. *Portulaca* can tolerate moderate to high salt concentrations and produce a considerable amount of dry mass even at relatively high salinities compared to standard crops [37,38]. Albeit the relative salt tolerance of other species in this genus has not yet been adequately studied.

The main objective of this research was to investigate the responses to salt stress of three different species of *Portulaca* (*P. oleracea* L. subsp*. oleracea*, *P. grandiflora* Hook. and *P. halimoides* L.) and a purslane cultivar (*P. oleracea* var. “Toucan Scarlet Shades”), under controlled experimental conditions, trying to specify the mechanisms of salinity tolerance in this genus promoting thus their use in saline agriculture and sustainable development, as a source of plant nutrients or ornamental species.

## 2. Results

### 2.1. Electrical Conductivity of the Substrate

Electrical conductivity (EC_1:5_) values in control soil samples varied between 0.55–1.08 dS m^−1^ throughout the treatment period. When salt stress was applied, EC increased in parallel to time (as salt accumulated in the substrate) and in a concentration dependent manner (400 mM registering the highest measured EC), with approximately 3, 4.5 and 7.5 dS m^−1^ in 100, 200 and 400 mM soils, respectively at the end of the treatments. It must be noted that differences in between the substrates of stressed plants of the 4 studied genotypes undergoing the same level of salt concentration treatment were statistically insignificant.

### 2.2. Growth Parameters

**Figure 1:**
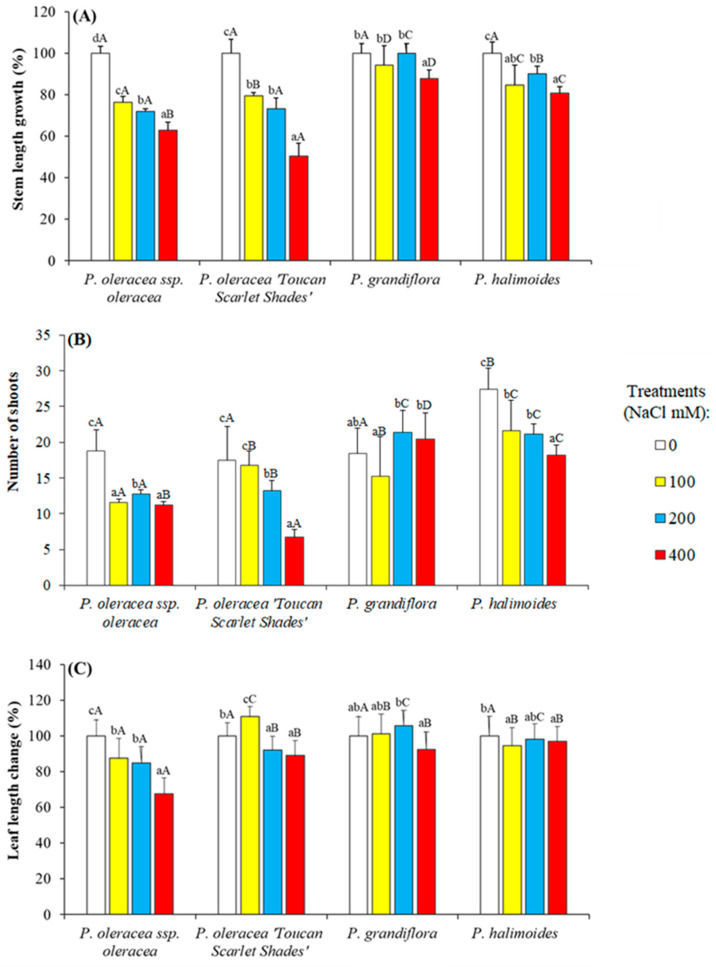
Measured growth parameters in the four studied *Portulaca* genotypes after 5 weeks of applied salt stress. (**A**) Stem length growth (%). For each genotype, values (means ± SE) are shown as percentages of the mean stem length of the control plants, considered 100%, (**B**) number of developed shoots by time of harvest, and (**C**) leaf length change (%). For each genotype, values (means ± SE) are shown as percentages of the mean leaf length of the control plants, considered 100%. Different lowercase letters within each genotype indicate significant differences among the treatments and different capital letters indicate significant differences among the genotypes undergoing the same treatment according to Tukey’s HSD test (*P* ˂ 0.05). Absolute values for the controls’ stem length are: 31.00 ± 1.04 cm (*P. oleracea* L. subsp. *oleracea*), 32.25 ± 2.20 cm (*P. oleracea* “Toucan Scarlet Shades”), 19.60 ± 0.92 cm (*P. grandiflora*) and 18.20 ± 0.96 cm *(P. halimoides*). Absolute values for the controls’ leaf length are: 4.00 ± 0.22 cm (*P. oleracea* L. subsp. *oleracea*), 2.93 ± 0.17 cm (*P. oleracea* “Toucan Scarlet Shades”), 2.6 1 ± 0.12 cm (*P. grandiflora*), 3.36 ± 0.09 cm) and 1.13 ± 0.05 cm *(P. halimoides*).

Our findings show that plant growth and productivity were adversely affected in all the four *Portulaca* genotypes studied, though in varying degrees as presented in Figure 1.

Stem lengths at time of harvest (5 weeks) for controls ranged from 17.37 cm to 31.00 cm in *P. halimoides* and *P. oleracea L*. subsp. *oleracea*, respectively. Applied soil salinity had a detrimental impact on the main stem growth of all four investigated genotypes, inducing a decrement in lengthening in parallel to increasing applied salt concentration. This varied between the different taxa, being very acute in the two studied *P. oleracea* genotypes, where stem length growth was reduced by nearly 40% and 50% in *P. oleracea* L. spp. *oleracea* and *P. oleracea* “Toucan Scarlet Shades”, respectively (Figure 1A). On the other hand, *P. grandiflora*, and *P. halimoides* seemed to be less affected with all applied salt concentrations, reporting a mere decrease in stem growth of 12.24% and 19.23%, respectively, in 400 mM NaCl treated plants.

The number of lateral shoots was significantly lower in three of the studied genotypes under applied salt conditions in comparison to their corresponding controls (all but *P. grandiflora*) (Figure 1B). The highest averaged shoot number was 27.4 in *P. halimoides*, while the lowest 17.5 was reported in *P. oleracea* “Toucan Scarlet Shades” (Figure 1B). The most sensitive to the highest applied salt concentration was *P. oleracea* “Toucan Scarlet Shades” reporting a decrease of 61.5% in shoot number during the salt treatments in comparison to its controls. On the other hand, the least affected accession in shoot number was *P. grandiflora* which showed an increase in shoot formation under 400 mM NaCl. Another monitored growth parameter was leaf length. Here again the effect of applied salt stress for 5 weeks, ranged from severe (causing a reduction by 35% relative to its controls leaf length) in *P. oleracea* L. spp. *oleracea* and mild (reporting a decrease from control leaf length by a mere 3% under 400 mM NaCl) in *P. halimoides* (Figure 1C). The other two genotypes, *P. oleracea* “Toucan Scarlet Shades” and *P. grandiflora* did not show significant changes in their leaf length neither under 100 and 200 mM NaCl nor under 400 mM NaCl treatments (10% reduction in comparison to their controls).

**Figure 2:**
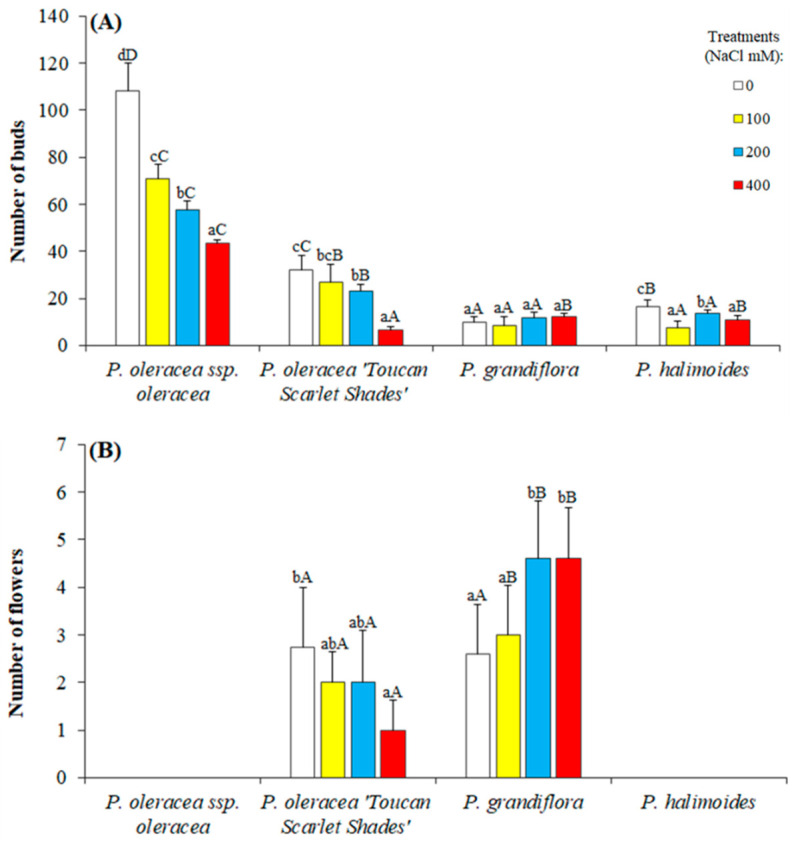
The influence of applied salt stress on the reproductive development of the four studied *Portulaca* genotypes. (**A**) Number of flower bud occurrence and (**B**) number of flowers. Different lowercase letters within each genotype indicate significant differences among the treatments and different capital letters indicate significant differences among the genotypes undergoing the same treatment according to Tukey’s HSD test (*P* ˂ 0.05).

Flower bud formation and flowering differed in unstressed plants among the four studied genotypes. *P. oleracea* L. subsp. *oleracea* produced the highest number of formed flower buds (about 108) (Figure 2A) though none developed into flowers (Figure 2B). On the other hand, only *P. oleracea* “Toucan Scarlet Shades” and *P. grandiflora* had any flower formation, where interestingly the latter had a higher number of flowers under applied salt stress. The number of formed flower buds decreased significantly in all investigated genotypes except in *P. grandiflora* where again it seems that the applied stress induced reproductive development with a 25% increase under 400 mM NaCl in comparison to control. 

**Figure 3:**
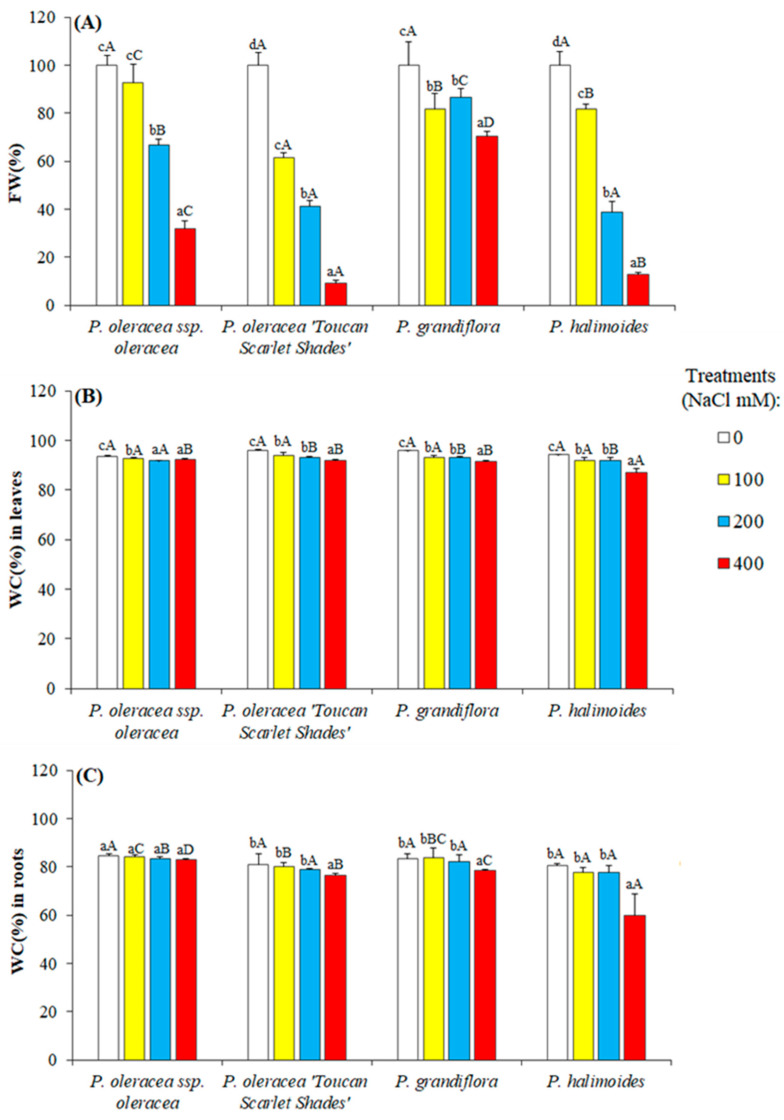
Total fresh yield and water content reduction in leaves and roots after 5 weeks of applied stress on four *Portulaca* genotypes. (**A**) fresh weight percentage are shown as percentages of the mean fresh weight of the control plants, considered as 100% (absolute values FW values in grams are 62.56 ± 5.82 g for *P. oleracea* L. subsp. *oleracea*, 112.00 ± 13.47 g for *P. oleracea* “Toucan Scarlet Shades”, 20.53 ± 4.43 g for *P. grandiflora*, and 11.85 ± 1.54 for *P. halimoides*), (**B**) water content percentage in the leaves (WC%), and (**C**) water content percentage in the roots. Different lowercase letters above the bars within each accession indicate significant differences among treatments and different capital letters denote significant differences among the accessions undergoing the same treatment according to Tukey’s HSD test (*P* < 0.05).

Upon completion of the applied salt treatments, plants were harvested and roots and shoots were weighted. A fraction of material was used for water content determination. Fresh weight in all studied taxa was dramatically reduced under the applied stress conditions (Figure 3A), displaying a reduction of almost 90% under 400 mM NaCl, compared to their respective controls for both *P. oleracea* “Toucan Scarlet Shades” and *P. halimoides*. On the other hand, fresh weight was reduced by almost 30% for *P. oleracea* L. subsp. *oleracea* under similar conditions, while *P. grandiflora* had the best resistance as it maintained 70% of its control FW when exposed to the highest stress treatment applied.

After oven drying the leaves, water content was calculated from the measured dry weight and afore measured leaf fresh weight for every harvested plant. Interestingly, water content in leaves and roots recorded only a small decrease in comparison to the loss in FW (Figure 3A–C). All studied genotypes showed only a small decrease in their roots’ WC under salt stress (Figure 3C). Only *P. halimoides* showed a significant decrease in its root’s WC when 400 mM NaCl was applied, showing a decrease to 60% from 80% measured in its unstressed plants.

### 2.3. Photosynthetic Pigments

Chlorophyll levels decreased in some of the studied genotypes under stress (Table 1), notably *P. oleracea* L. subsp. *oleracea* and to a lesser extent in *P. grandiflora*. Furthermore, an increase in these pigments was registered in *P. oleracea* “Toucan Scarlet Shades” and *P. halimoides*. Carotenoids contents increased under 400 mM NaCl in all studied genotypes except *P. oleracea* L. subsp. *oleracea* where it nearly remained equal to that measured in its control plants (Table 1). In terms of carotenoids’ increase, *P. halimoides* reported the biggest increment (2.71-fold change as compared to its control), followed by *P. oleracea* “Toucan Scarlet Shades” (1.35-fold change).

### 2.4. Osmolytes

**Figure 4:**
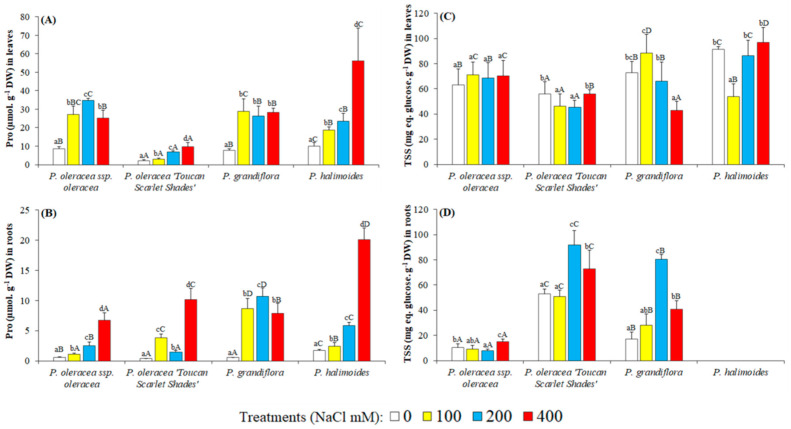
Osmolytes quantified in the four *Portulaca* genotypes subjected to salt stress treatments. Proline (Pro) and total soluble sugars (TSS) accumulation in the leaves (**A**,**C**, respectively) and roots (**B**,**D**, respectively). The values shown are means ± SE (*n* = 5). Different lowercase letters above the bars indicate significant differences between the control plants, and different capital letters indicate significant differences between different genotypes.

The findings of the current research show that proline (Pro) content of the leaves in the control *Portulaca* genotypes were relatively low ranging from 2.19 μmol g^−1^ DW (*P. oleracea* “Toucan Scarlet Shades”) to 8.50 μmol g^−1^ DW (*P. oleracea* L. subsp. *oleracea*) (Figure 4A). Similarly, Pro levels were also low in the roots, ranging from 0.35 μmol g^−1^ DW (*P. oleracea* “Toucan Scarlet Shades”) to 1.6 μmol g^-1^ DW (*P. halimoides*) in control plants (Figure 4B). Proline accumulation was found both in leaves and roots of the plants subjected to salt stress treatments. After 5 weeks of applied stress, Pro accumulation in the leaves was the highest in *P. halimoides* with a 5.66-fold change increase, and similarly a 12-fold increment from control levels in their roots. In addition, *P. oleracea* “Toucan Scarlet Shades” leaves registered the smallest increase under stress (a mere 9.67 μmol g^−1^ DW in 400 mM NaCl stressed plants). Therefore, a positive correlation was shown between proline level and salt treatment intensity; however, proline accumulation was not also related to the degree of salt tolerance in these species (Figure 4A,B).

Among the tested *Portulaca* genotypes, the soluble sugar content of the leaves in the untreated controls varied from 46 (*P. oleracea* “Toucan Scarlet Shades”) to 91 (*P. halimoides*) mg eq. glucose g^−1^ DW (Figure 4C). Under salt stress conditions no clear correlation was shown between TSS content and salt stress intensity. In the leaves, TSS followed different accumulation patterns: (i) Soluble sugar level increased at 100 mM NaCl concentration, followed by a decrease at 200 mM NaCl and increasing again at the highest salt concentration (e.g., *P. oleracea* subsp. *oleracea*), (ii) soluble sugar level increased at 100 mM NaCl concentration but decreased at 200 and 400 mM NaCl concentrations (e.g., *P. grandiflora*) and (iii) TSS content decreased at the lowest salt concentration but increased at 200 and 400 mM NaCl concentrations (*P. oleracea* “Toucan Scarlet Shades” and *P. halimoides*). *P. grandiflora* showed a significant decrease (41.20%) in soluble sugar content at the highest salinity level as compared to control (42.93 mg eq. glucose g-1 DW vs. control 73.024 mg eq. glucose g^−1^ DW). 

In the roots, a variable pattern of TSS content was found as the salt concentrations increased. It was observed that the TSS content of all genotypes was significantly higher at the highest salt concentrations applied than in controls. Statistically significant differences were recorded at 200 and 400 NaCl concentrations as compared to controls (Figure 4D). No data was obtained in *P. halimoides* due to an accidental loss of material during lab work.

### 2.5. Malodialdehyde and Non-Enzymatic Antioxidants

**Figure 5:**
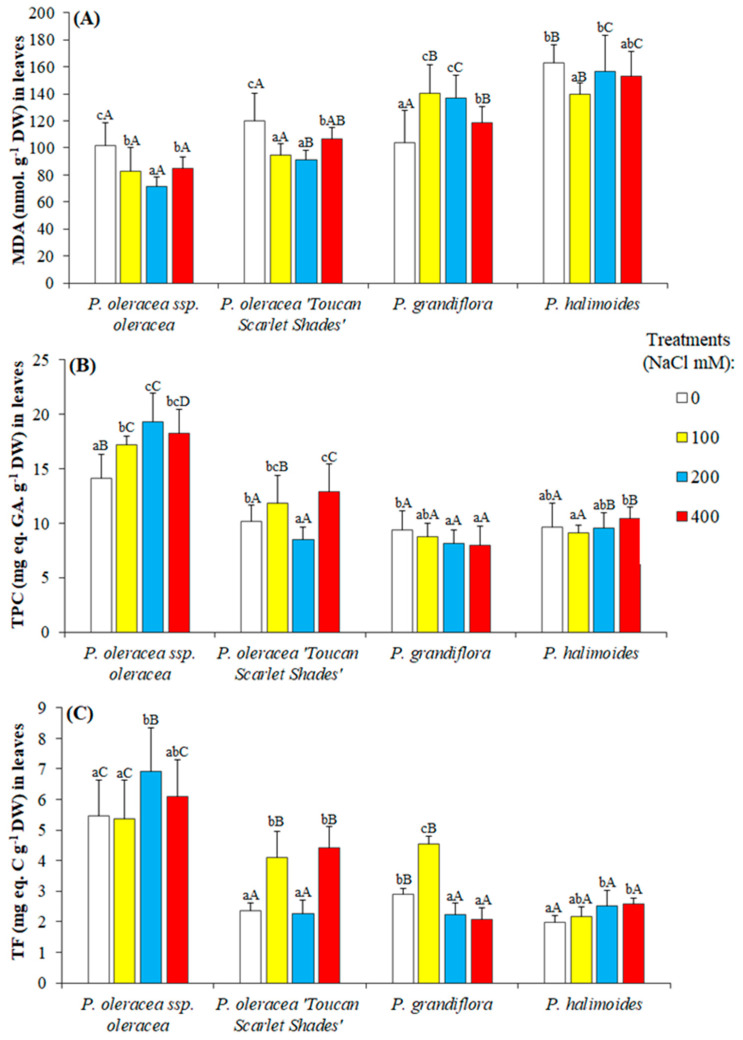
Malondialdehyde (MDA) and chemical antioxidants accumulation in the leaves of *Portulaca* genotypes after 5 weeks of salt stress treatments. (**A**) MDA, (**B**) total phenolic compounds (TPC), and (**C**) total flavonoids (TF). The values shown are means ± SE (*n* = 5). Different lowercase letters above the bars indicate significant differences between the control plants, and different capital letters indicate significant differences between different genotypes.

After 5 weeks of salt treatment, it was expected that the MDA in the leaves of stressed plants would show an increase consistent with the increased salinity level, but in the *Portulaca* genotypes investigated, this was not the case, (Figure 5A), as MDA levels in the leaves fluctuated under applied stress. 

The pattern of variation of total flavonoid content in response to increasing salt concentrations was very similar to that of TPC (Figure 5B,C). Among the untreated control plants, it was observed that flavonoid content varied, with the highest content in *P. oleracea* L. subsp. *oleracea* (5.45 C. g^−1^ DW), while the lowest was in *P. halimoides* L. (1.97 C. g^−1^ DW). Under applied salt stress, TPC and TF levels fluctuated in the leaves of all four studied genotypes, showing no strong correlation with the increasing concentrations of applied NaCl, except for *P. oleracea* L. subsp. *oleracea*, though *P. oleracea* “Toucan Scarlet Shades” reported a significant increment in its accumulated TPC and TF when 400 mM NaCl was applied (in comparison to its controls).

### 2.6. Ionic Content

**Figure 6:**
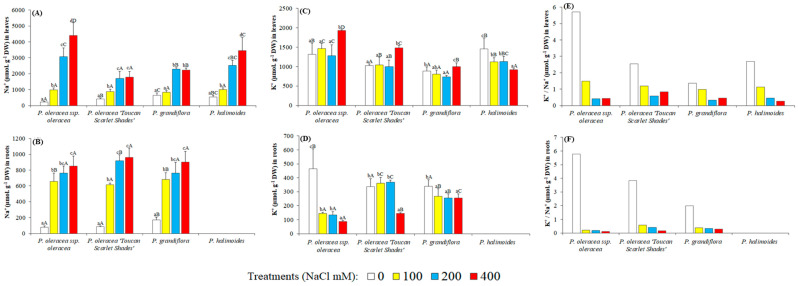
Ion content of leaves and roots of the 4 studied *Portulaca* genotypes undergoing 5 weeks of salt treatments (up to 400 mM NaCl). Sodium and potassium concentrations, as well as potassium over sodium ratios in leaves (**A**,**C**,**E**, respectively and roots (**B**,**D**,**F**, respectively). The values shown are means ± SE (n = 5). Different lowercase letters above the bars indicate significant differences between the control plants, and different capital letters indicate significant differences between different genotypes.

Salinity induced the accumulation of Na^+^ and Cl^−^ in high amounts in roots and leaves of the four genotypes in response to salt stress acting as Na^+^ and Cl^−^ includers. Sodium levels in NaCl-treated plants of all genotypes showed an applied-salt-concentration-dependent increase (Figure 6A,B). It increased up to 4.5 mmol g^−1^ DW in the leaves of 400 mM NaCl stressed plants of *P. oleracea* L. subsp. *oleracea* and *P. halimoides*, while *P. oleracea* “Toucan Scarlet Shades” and *P. grandiflora* reported merely half those levels under similar conditions (Figure 6A). Control contents of sodium in the leaves of studied plants were similar in all four genotypes, and the increase was comparable when 100 and 200 mM NaCl were applied. Interestingly however, was the trend in chloride ions accumulation in the leaves, which were expected to mimic that of sodium. *P. grandiflora* reported chloride levels 4 folds lower than that of *P. oleracea* L. subsp. *oleracea* and nearly 2.5 times that of *P. halimoides* and *P. oleracea* “Toucan Scarlet Shades” (Table 2). Chloride accumulation in the leaves seemed to reach a plateau after 100 mM NaCl in *P. grandiflora* and 200 mM in *P. halimoides*. As for potassium in the leaves (Figure 6C), all studied genotypes reported an increment under 400 mM NaCl, except for *P. halimoides* which showed a 30% decrement. When correlating the aforementioned data through K^+^/Na^+^ ratios, *P. oleracea* L. subsp. *oleracea* had the biggest decrease though it had the highest ratio in non-stressed conditions (Figure 6E). Moreover, *P. oleracea* “Toucan Scarlet Shades” and *P. grandiflora* had the smallest decrease in this ratio, with the latter retaining the smallest K^+^/Na^+^ ratio in control conditions.

In the roots, sodium contents increased under applied salt stress to threshold limits than that of stems (Figure 6B), up to about 1 mmol g^−1^ DW in all studied genotypes under 400 mM NaCl (the roots of *P. halimoides* were not investigated due to the aforementioned reasons). The most uniform decreasing pattern in K^+^ content was observed at root level (Figure 6D); the highest reduction percentage as compared to control plants was recorded in *P. oleracea* L. subsp. *oleracea* (80.94%) contrary to *P. grandiflora* with only a 24.5% reduction when compared to untreated control plants. This translates to the biggest drop in K^+^/Na^+^ ratios being in *P. oleracea* L. subsp. *oleracea* again (Figure 6F). Chloride contents increased in parallel to applied salt in all genotypes, showed the highest accumulation in *P. oleracea* L. subsp. *oleracea* (Table 2).

### 2.7. Principal Component Analysis

**Figure 7:**
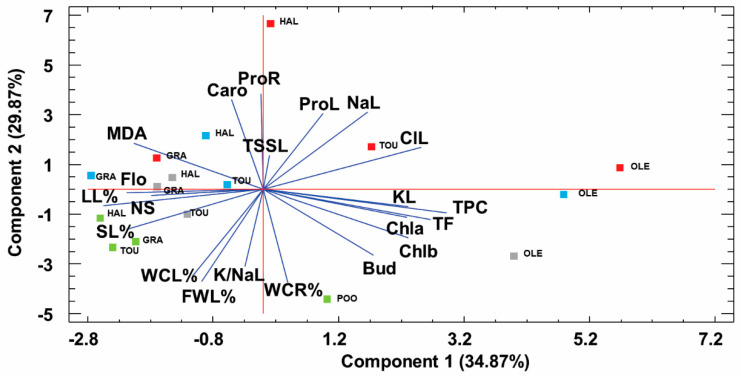
Principal component analysis based on the morphological and biochemical traits measured in plants from control (green), 100 (grey), 200 (blue) and 400 mM (red) NaCl treatments of *Portulaca oleracea* susbp. *oleracea* (OLE), *P. oleracea* cultivar “Toucan Scarlet Shades” (TOU), *P. grandiflora* (GRA) and *P. halimoides* (HAL). Abbreviations of the analyzed parameters: SL%, stem length percentage (control being 100%); NS, number of stems; LL%, leaf length percentage (control being 100%); flower bud, number of flower buds; Flo, number of flowers; FWL%, fresh weight percentage of leaves (control being 100%); WCL%, water content percentage in leaves; WCR%, water content percentage in roots; ProL, proline in leaves; ProR, proline in roots; TSS, total soluble sugars in leaves; MDA, malonaldehyde; TPC, total phenolic compounds; TF, total flavonoids; CL, chloride in leaves; NaL, sodium in leaves; KL, potassium in leaves; K/NaL, potassium over sodium ratio in leaves.

A principal component analysis was performed with all analyzed parameters (except those of total soluble sugars and ions in roots due to the incomplete data set in *P. halimoides*). Five components were found with an Eigenvalue higher than 1, with their cumulative weight accounting for almost 90% of the total variability. The first component, explaining 34.87% of the variability was positively correlated with the levels of total phenolics (TPC) and flavonoids (TF), and K^+^ in leaves, and to lesser extent to those of chlorophyll a (Chla) and (Chlb) and negatively with morphological parameters such as length of the leaves (LL), number of shoots (NS), number of flowers (Flo), and stem length (SL) (Figure 7). The second component explaining an additional 29.87% of variability was positively correlated with levels of osmolytes: Proline in roots (ProR) and leaves (ProL), and total soluble sugars in leaves (TSSL), and carotenoids (Caro) and negatively with the water content of roots (WCR) and leaves (WCL), fresh weight of leaves (FWL) and the ratio K^+^/Na^+^ in leaves (Figure 7). Regarding the projections of the genotypes on the scatterplot, *P. oleracea* susp. *oleracea* (OLE) separated from the other three analyzed, mostly due to high content of TP, TF and K^+^ in the salt-stressed plants. Controls of the other three taxa are grouped together. Interestingly, plants from the most stressful treatment of 400 mM NaCl were separated along the first axis, except those of *P. halimoides* (HAL), which was clearly related to the second component. On the other hand, only *P. grandiflora* (GRA) was projected on the left side of the graph together, related with higher leaf length (LL), number of shoots (NS) and flowers (Flo), indicating a better response to salinity (Figure 7).

## 3. Discussion

Purslane (*P. olereacea*) has been proposed as an ideal candidate to be used as a halophytic crop with high nutritional value in the drainage water reuse system [39], and it was reported to accumulate higher biomass under mild salt concentrations and complete its life cycle even at a high salinity level (approximately 350 mM NaCl), maintaining its shoots free of visible salinity induced injury or nutrient deficiency symptoms [39]. This species was reported as moderate salt tolerant in many other studies [40,41,42]. Besides the accumulation of Na^+^ and Cl^−^, there are reports regarding its ability to accumulate higher levels of K^+^ in shoots in conditions of salt stress, reflected in the K^+^/Na^+^ selectivity coefficients [39]. Due to its salt tolerance and edible properties purslane was regarded as a good candidate for soil desalinization [43,44], and given its potential in retaining toxic ions, also in heavy metals decontamination [45,46,47]. In a recent metabolomics study performed on two cultivars of purslane, 132 different metabolites were marked for their significant variation under salinity, notably an increase in proline and other amino acids, neutral and soluble sugars, sugar alcohols, amines, etc. [48]. Growth of both cultivars was strongly affected by the salinity, starting with 100 mM NaCl but metabolic response of the cultivars differed. Also, strong differences in growth parameters, flowering, mineral content or mechanism of salt tolerance were reported when comparing different genotypes of *P. oleracea* [49,50]. In a study analyzing responses to salt stress in a wild and a cultivated variety of *P. oleracea*, simultaneous changes in multiple traits indicated that the two genotypes employed different strategies in allocating resources to cope with saline stress [51]. This great phenotypic plasticity makes the species, and at a larger extent the genus *Portulaca* as a whole ideal for comparative studies focused on the better understanding of mechanism of stress tolerance [52].

Contrary to purslane, which was extensively analyzed in recent years, due to its interest as crop and medicinal plant, only little is known on the other two species included in the study. *P. grandiflora* has been reported as salt tolerant and as an accumulator of proline [53]. Recently, in addition to the C4 photosynthesis common in the genus *Portulaca*, the pathway CAM was found in cotyledons of this species [54]. According to our knowledge, there are no studies regarding the salt tolerance of *P. halimoides*, except our previous reports on germination and early seedling growth in *P. oleracea*, *P. grandiflora* and *P. halimoides* [55] which revealed a greater resilience to drought of the latter, and a seemingly better response to salinity in *P. grandiflora*.

The data presented in this work confirm the salt tolerance of purslane and of congener species. The four *Portulaca* genotypes survived 5 weeks of salt treatments even when 400 mM NaCl was applied. However, growth was affected which was expected since the most common effect of salinity is the inhibition of plant growth [13]. Growth inhibition under stress conditions is strongly related to the redirection of plant resources such as energy and metabolic precursors from their primary metabolisms and growth for the activation of defense mechanisms [14]. Although all *Portulaca* genotypes followed the same pattern of growth response, the smallest reduction of all growth parameters was observed in *P. grandiflora*, which at the end of the salt treatments showed a slight increase of the leaf length and only a small reduction of the shoots fresh weight and water content. This can be explained by a decrease in growth in terms of dry matter while the plants have retained their water content notably in the leaves, around 90% even under the highest applied stress (Figure 3B). This good water retention in the leaves could be explained by the constitutive succulence of the leaves of the *Portulaca* species. Succulence in combination with other adaptive traits, such as reduced leaf surface, leaf thickness, increased cell wall plasticity, or a small number of stomas per unit area, is considered one of the defining features of halophytes as an adaptive structural trait. In addition, the succulence has the effect of diluting the salts that can accumulate in the plant’s organs, which allows the plant to cope with a large amount of salts [56]. Previous reports suggest that the osmotic potential of the sap from the leaves of plants grown under saline conditions can change to maintain a constant water uptake from the soil, based also on the osmotic adjustment at the root level [57,58,59]. A better response in terms of growth parameters to salt stress of this *P. grandiflora*, especially to the highest concentration of 400 mM NaCl, was also reflected in the scatter plot of the principal component analysis. On the other hand, it was observed that relatively small supplements in salt concentrations were enough to reduce vegetative growth and plant development in some genotypes (*P. oleracea* L. subsp. *oleracea* and *P. halimoides*). Greater salt tolerance of *P. grandiflora* was highlighted also by its flowering rate under salt treatments. 

Applied salinity is known to have deleterious effects on the photosynthetic pigments in stressed plants, due to activity of ROS which accumulate due to oxidative stress resulting from ionic toxicity and osmotic imbalance [60]. A reduction in the photosynthetic pigments is considered a frequent response of the plants encountering stressful environments [61] being negatively correlated with their relative degree of tolerance to stress conditions [62]. Remarkable decreases in chlorophyll content as a result of drought and salt stress have been observed in various plant species including both trees, and herbaceous species, such as *Paulownia imperialis* [63], *Phaseolus vulgaris* [64], *Carthamus tinctorius* [65], *Salicornia prostrata* [66] or *Inula crithmoides* [67] but with some exceptions among halophytes including *Tecticornia indica* [68], *Sesuvium portulacastrum* [68], *Salvadora persica* [69], *Sarcocornia fruticosa* [70]. In the latter it has been observed that under saline conditions the leaf pigment content varied; in some species the total chlorophyll content was significantly enhanced exhibiting a gradual salt-dependent increase (*T. indica* and *S. portulacastrum*) whereas in others (*S. persica* or *S. fruticosa*) no significant changes were evidenced in chlorophyll a, b, total chlorophyll, and carotenoid contents with increasing salinity [69].

Previous reports indicate that purslane accessions subjected to salt stress generally showed a reduction of their chlorophyll concentration but the relative reductions with respect to the non-stressed controls were maintained below 40%, even at 32 dS m^−1^, the highest salinity tested [71]. Our findings are in accordance with previous results indicating that a decrease in chlorophylls in some studied genotypes under stress, notably *P. oleracea* L. subsp. *oleracea* and to a lesser extent in *P. grandiflora*. On the other hand, an increase in these pigments was registered in *P. oleracea* “Toucan Scarlet Shades” and *P. halimoides* which is not unusual in plants undergoing low to medium-level stress, as a requirement to enhance the capacity for stress-defense. However, this precedes the expected degradation in case these unfavorable conditions are prolonged [72]. Carotenoids contents increased under the highest concentration of NaCl applied in all studied genotypes except *P. oleracea* subsp. *oleracea*. Some carotenoids such as xanthophylls and β-carotenes are well-known for their radical quenching activities, rendering them invaluable for photoprotection against accumulating ROS [73]. 

Growth parameters are important stress indicators used to evaluate the impact of different stresses on plants; but when they are combined with “stress biomarkers” which are associated with specific plant responses, they can provide useful information about tolerance mechanisms. 

Prior investigations carried out in different plant species confirm that the accumulation and increased production of proline is correlated with stress tolerance. In transgenic tobacco plants, increasing proline content led to an enhanced resistance to drought and salinity [74]. These transgenics over-expressed the P5CS gene—encoding the enzyme that controls the rate-limiting step of proline biosynthesis from glutamate. However, proline accumulation is not always positively correlated with stress tolerance, but could also serve as a symptom of injury in leaves caused by salt [75]. Such were the results obtained especially in food crops rather than halophytes. A previous report reveals that sorghum genotypes differed in their sensitivity to salt [76]. Other studies indicate a negative correlation of proline accumulation with tolerance; for example, in two *Phaseolus* sp. the highest concentrations were measured in the most sensitive genotypes [77]. Similar results have been reported in maize, where proline accumulation in the salt tolerant genotypes (*Zea mays* L. cv. Ceratina) was significantly lower than that in the salt sensitive one (*Zea mays* L. cv. Sacharata) as well as in rice (*Oryza sativa* L.) with lower levels in cultivar IR28 (salt susceptible) than in Pokkari (salt tolerant) [78,79]. The results reported in this work indicate that the concentration of proline in the genotypes analyzed could not be related to their salt tolerance. Although, proline levels significantly increased in all selected genotypes in salt stressed plants, the smallest increment was registered in the most least damaged genotype, *P. grandiflora*. 

The accumulation of total soluble sugars (TSS) in plants has been reported to play a role in response to abiotic stresses in many plant species [80]. The role of soluble sugars might be masked by their additional roles in plants as being products of the photosynthesis or components of primary metabolisms, signaling or regulatory networks which make sometimes difficult to clearly define their specific contribution to stress tolerance [81]. In the current research, levels of TSS increased only slightly in response to salinity, or their concentrations even decreased. As such, it is difficult to assess their role in salt tolerance of the analyzed taxa.

As reported for many other plant species, MDA is a product of membrane lipid peroxidation which is considered as a reliable biochemical oxidative stress marker; high MDA level should be correlated with high degree of oxidative stress [82]. However, our results show that MDA levels exhibited only minor variations that could not be correlated with the concentration of salt applied or with the degree of salt tolerance of the genotypes. This reflects a low to no oxidative stress among the investigated four genotypes. It can be concluded that lipid peroxidation due to oxidative stress could not occur within such a short period of applied stress (5 weeks).

Phenols and flavonoids are secondary metabolites which act as antioxidants, and their accumulation in plants can reduce the oxidative damage caused by abiotic stresses [83]. Total phenolic compounds (TFC) and total flavonoids (TF) increased significantly under salt stress mostly in *P. oleracea* subsp. *oleracea*, which also contained high concentrations in control plants, and to a lesser extent in its cultivated variety “Toucan Scarlet Shades”, but not in the other two species analyzed.

High level of salt stress alters homeostasis in water potential and ion distribution. Drastic changes caused by the accumulation of Na^+^ and Cl^−^ lead to molecular damage, growth arrest and even plant death due to the induced cytoplasmic toxicity [84]. To achieve tolerance against stress plants are controlling homeostasis by regulating ion transport within the plant, cellular uptake and intercellular distribution of Na+ and other toxic ions. Glycophytes activate mechanisms to reduce the uptake of Na+ and Cl-at root level or block their transport to the aerial parts of the plant, whereas dicotyledonous halophytes accumulate and sequester toxic ions in their vacuoles [84]. Our results show that Na^+^ concentrations increased with to the concentration of NaCl applied and were significantly higher in the leaves of the selected *Portulaca* genotypes than in their roots, indicating that they act as sodium accumulators. In general Na^+^ accumulation is associated with a decrease of K^+^ levels since these two cations are competing for the same binding sites. A significant reduction of K^+^ was observed in the roots of all plants from the 400 mM NaCl treatment, but this was associated with an increase of its levels in leaves, indicating that the active transport from roots to leaves is as essential mechanism of salt tolerance in the analyzed *Portulaca* genotypes. The low accumulation of Na^+^ in leaves associated with only a small reduction of the ratio between K^+^ and Na^+^ in leaves, and a small decrease of K^+^ in roots in plants of *P. grandiflora* submitted to 400 mM NaCl concentration, are the key elements explaining its better adjustment to high salinity. *P. grandiflora* was also the species with the lowest accumulation of Cl^−^ in roots and leaves under higher salt concentrations.

## 4. Materials and Methods

### 4.1. Plant Material and Stress Treatments

Three species of *Portulaca* were included in the study, purslane (*P. oleracea* L), moss rose (*P. grandiflora* Hook.) and silkcotton purslane (*P. halimoides* L.). Of the first species, two genotypes were compared, the wild *P. olereacea* subsp. *oleracea* and the cultivar *P. oleracea* “Toucan Scarlet Shades”. Seeds used in this experiment were received from different Botanical Gardens (Botanicher Garten der Universität Zürich, Botanischen Garten der Universität Potsdam, Hortus Botanicus Bergianus—Bergianska trädgården, Grădina Agro-Botanică USAMV Cluj-Napoca) based on the Agreement on the supply of living plant material for non-commercial purposes while the ornamental variety was purchased from a commercial supplier from the USA.

Seeds of the four *Portulaca* genotypes (*P. oleracea* L. *subsp. oleracea*, *P. grandiflora* Hook., *P. halimoides* L. and *P. oleracea* “Toucan Scarlet Shades”) were germinated in growth chambers under a photoperiod of 16 h, and a temperature of 25 °C. After 10 days of growth the seedlings were transferred individually to 0.5 L pots (9 × 9 × 10 cm) with a nutritive mix of peat:vermiculite:perlite (50:25:25) substrate. The plants were grown in the greenhouse of the Institute for Plant Molecular and Cellular Biology, Valencia, Spain (39°28′43.0″ N 0°20′12.1″ W) under the following conditions: Long-day photoperiod (16 h light/8 h dark), with light intensity of 130 μE m^−2^ s^−1^, temperature (23 °C during the day and 17 °C at night), Humidity ranged between 50–80% during the time course of the treatments. During the growing period, seedlings were watered twice a week by adding 1.5 L by half-strength Hoagland’s nutrient solution [85] to each tray (containing 12 pots).

Salt treatments were started after six weeks of growth [86] and applied to nine selected seedlings with homogeneous growth stage per treatment. During salt treatments, plants were treated twice a week with newly-prepared nutritive solutions supplemented with respective salt concentrations (100 mM, 200 mM and 400 mM), applying 150 mL solution for each pot. To avoid osmotic shock of salinity, the watering solution was added to the bottom of the trays providing thus a gradual absorption of the solution. In the meantime, control plants were irrigated in the same regime barring any added salt. Plants were harvested after 5 weeks of treatments. Plant roots, stems, and leaves were sampled separately at harvest.

Electrical conductivity (EC) of the soil (pot substrate) was measured at the beginning (Control) and at the end of the treatments applied to the four studied *Portulaca* genotypes. Soil samples were taken from three different pots of every treatment, air dried, diluted and then passed through a 2-mm opening sieve. The extract ratio used for determining EC was 1:5 soil:water mixture. The suspension was prepared in Milli Q water and stirred for 60 min at 600 u/min at 23 °C. Electrical conductivity was measured with a Crimson Conductometer 522. The values reported were expressed in dS m^−1^.

### 4.2. Plant Growth Parameters

To determine the effects of applied salinity on plant growth, five plants per treatment per studied accession were monitored for the following physiological parameters: (a) Stem length, (b) leaf length, (c) number of stems and shoots, (d) number of flower buds and flowers, (e) dry weight and (f) water content.

The length of the main stem was measured every five days during the applied treatment period. The average of the five studied plants per stress treatment for every investigated accession was calculated, before being converted into percentage relative to the observed average measured for each species/cultivar’s control at that time point.

Similarly, the length of five leaves per plant was measured for the same aforementioned plants per treatment for each studied accession. The leaves were chosen randomly from three different parts (layers) of the seedling: Lower, middle and upper part. The mean length of the leaves (in cm) was then calculated, and expressed as percentages of the average leaf length of the corresponding non-stressed control, taken as 100%. The total number of shoots per plant developed under stressed and unstressed conditions was recorded at the end of the treatments. In order to evaluate the reproductive traits, the flower bud occurrence was monitored during the treatments. The total number of flower buds and flowers for each treatment were averaged.

In addition to the above-mentioned growth parameters measured during the treatments, stress-induced inhibition of plant growth was also assessed by determining the mean fresh weight (FW), dry weight (DW) and water content (WC) of the leaves after 5 weeks of applied salt stress treatment. The fresh weight (FW) of the plants was measured separately for leaves and roots. After harvest, fresh plant material from both harvested tissues from all genotypes was weighed. The mean was then calculated and expressed in percentages (relative to each genotype’s respective control, serving as 100%). After measuring the fresh weight, a part of the fresh material was dried in the oven for four days at 65 °C, until constant weight in order to obtain DW. The fresh weight and dry weight data were then used to calculate the water content percentage of the three harvested tissue per studied plant according to the following formula [87]:

WC (%) = [(FW − DW)/FW] × 100

### 4.3. Biochemical Plant Responses to Salt Stress

After plant harvest, biochemical analyses were carried out to assess the salinity-induced changes in the levels of different biochemical stress markers including: Photosynthetic pigments (Chlorophyll a and b and carotenoids), osmolytes (proline, total soluble sugars), malondialdehyde (MDA), total phenolic (TPC), flavonoids (TFC), and ionic content.

#### 4.3.1. Photosynthetic Pigments

Chlorophyll a (Chl a), chlorophyll b (Chl b), and total carotenoid (Caro) levels were determined via spectrophotometry, using 100 mg fresh leaf material from each of the four studied *Portulaca* genotypes. The collected fresh leaves were ground in a MM 301 mixer mill in the presence of liquid N_2_ and then extracted with 1 mL ice-cold 80% acetone. Samples were mixed in the dark overnight on a shaker at 4 °C, before being centrifuged at 4 °C for 15 min (3,000 rpm). The supernatant (100 μL) was transferred into new tubes and diluted with another 900 μL of ice-cold acetone. Samples were mixed thoroughly and absorbance was recorded at 663, 646 and 470 nm. Five replicates were used for each treatment. The amount of pigments in each sample was calculated according to the following equations (80% acetone was used as blank) [88]:

Chl ‘a’ (µg mL^−1^) = 12.21 (A663) − 2.81 (A646);

Chl ‘b’ (µg mL^−1^) = 20.13 (A646) − 5.03 (A663);

Chl ‘a’+‘b’ (µg mL^−1^) = 20.13 (A646) − 5.03 (A663). 

Caro (µg mL^−1^) = [1000 × A470 − (3.27×Chl ‘a’) − (104 × Chl ‘b’)]/227

Final values of the photosynthetic pigments were expressed as mg. g^−1^ DW.

#### 4.3.2. Osmolytes

Free proline (Pro) content was measured in fresh tissue from the leaves and roots according to the ninhydrin-acetic acid method of Bates et al. [89]. The frozen plant material was homogenized in 3% (*w/v*) aqueous sulfosalicylic acid, mixed with 1 mL acid-ninhydrin and 1 mL of glacial acetic acid. Samples were incubated in boiling water for 1 h at 95 °C. To stop the reaction, samples were cooled down in an ice bath and the reaction mixture was extracted with 2 mL toluene, mixed vigorously and left at room temperature for phase separation. The absorbance of the supernatant was measured at 520 nm using toluene for blank. Proline concentration was determined from a standard curve and expressed in μmol g^−1^ DW. Total soluble sugar (TSS) content of the *Portulaca* samples was quantified according to the method described by Dubois et al. [90]. Dried material was ground and mixed with 3 mL of 80% methanol on a rocker shaker for 24–48 h. The mixture was filtered and centrifuged at 13 000 rpm for 20 min. Concentrated sulfuric acid and 5% phenol was added to the samples and the absorbance was measured at 490 nm. Glucose solutions of known concentration were used to obtain a standard curve, and TSS contents were expressed as “mg equivalent of glucose” per gram of DW.

#### 4.3.3. Malondialdehyde

Lipid peroxidation levels in the leaves of the four *Portulaca* genotypes were determined measuring MDA content by using thiobarbituric acid-reactive substances (TBARS) assay, as described by Hodges et al. [91]. The ground dry material (0.05 g) was extracted with 400 μL of methanol 80%, by mixing in an orbital shaker for 12 h. After mixing, the sample was filtered through glass wool and then 800 μL of 20% TCA and 800 μL of 0.65% TBA were added to the samples which were mixed vigorously and heated at 95 °C in a block heater for 25 min, cooled and centrifuged at 3000× *g* for 10 min. The supernatant‘s absorbance was recorded at 440 nm, 532 nm and 600 nm using a spectrophotometer. The blank was prepared in the same way but without plant extract. MDA concentration was calculated from the absorbance at 440, 532 nm and measurements were corrected for non-specific absorbance by subtracting the absorbance at 600 nm. Malondialdehyde equivalents were expressed as μmol MDA g^−1^ FW.

#### 4.3.4. Total Phenolic and Flavonoid Content

Total phenolic compounds (TPC) were quantified in leaves of the four *Portulaca* genotypes by reaction with Folin–Ciocalteu reagent, according to Blainski et al. [92], using 50 μL of plant extract (extracted from dry matter in 80% methanol). and measuring the absorbance at 765 nm using a spectrophotometer. The amount of TPC was expressed as milligram of Gallic acid (used as standard) equivalents per g of dry weight of the sample (mg eq. GA g^−1^ DW). Total flavonoids (TF) were determined by using the aluminum chloride colorimetric assay according to Zhishen et al. [93] protocol. The absorbance of the samples was measured in comparison to the prepared blank reagent at 510 nm using a spectrophotometer. The total flavonoid content of the leaves was expressed in equivalents of catechin (mg eq. C g^−1^ DW), used as standard.

#### 4.3.5. Ion Content

To perform the ion content measurements, 0.05 g dried and ground plant material (leaves and roots) was suspended in 2 mL distilled water and homogenized. The supernatant was transferred to plastic tubes and boiled at 100 °C for 7 min. The samples were cooled down and centrifuged at 20,000× *g* for 10 min at 4 °C. The supernatant was transferred into a 1 mL syringe, and then passed through 0.22 µm filters. Na^+^, K^+^ levels were quantified by using a Flame Photometer Jenway PFP7 while Cl^−^ were measured using a potentiometer titrator—848 Titrino plus.

#### 4.3.6. Statistical Analyses

All data was analyzed using IBM SPSS Statistics 19 (IBM Corporation, Portsmouth, UK) and Statgraphics Centurion 18 (Statgraphics Technologies, The Plains, VA, USA). One-way analysis of variance (ANOVA) was assessed at 95% confidence level to determine whether there were any statistically significant differences between the means of the treatments within one genotype and for all the genotypes undergoing the same treatment. When the ANOVA null hypothesis was rejected, post-hoc comparisons were performed using Tukey’s honestly significant difference test at *P* < 0.05 to determine significant differences between the means. The values shown throughout this manuscript’s text, tables and figures, are means ± SE. Furthermore, all parameters recorded in plants from control and salt treatments were correlated by principal component analysis (PCA).

## 5. Conclusions

The testing of the interspecific variation in salinity in *Portulaca* genotypes allowed a better understanding of the essential mechanisms of salt tolerance in this genus. Based on sustained vegetative growth under applied salt stress, as a criterion for improved adaptability, *P. grandiflora* is assumed to be the least susceptible of the genotypes studied. However, contrary to previous reports in *Portulaca*, which considered proline accumulation as a key response to confer salinity tolerance, the present study revealed only a small increase in proline in the more tolerant *P. grandiflora*. Our study demonstrates that tolerance in the studied taxa depends rather on the maintenance of K^+^ homeostasis. When exposed to high salinity, all genotypes accumulated Na^+^ and Cl^−^ in the roots and leaves at substantial levels, acting as Na^+^ and Cl^−^ includers. The role of K^+^ in the maintenance of cellular functions is well documented and, as such, its sustained presence in these detrimental conditions ensures the continuation of enzymatic functions and the maintenance of the integrity of photosynthetic pigments, which guarantee greater tolerance. However, slight differences in ion transport have been observed between the four genotypes studied. The significant increase of Na^+^ in the leaves of *P. oleracea* subsp. *oleracea*, *P. oleracea* “Toucan Scarlet Shades” and *P. grandiflora* at the highest salt concentration applied and the decrease of the K^+^/Na^+^ ratio in leaves with increasing salinity suggest that Na^+^ was transported in greater proportion than K^+^ to this organ in these genotypes.

## Figures and Tables

**Table 1:** Photosynthetic pigments in the leaves of the four studied *Portulaca* genotypes. Chlorophyll a (Chl a), chlorophyll b (Chl b) and carotenoids (Caro) contents after 5 weeks of applied salt stress. Values shown are means ± SE. For each pigment, different lowercase letters in a column indicate significant differences between treatments; different capital letters in a row denote significant differences among the genotypes undergoing the same treatment.

Photosynthetic Pigment (mg g^−1^ DW)	Treatment (mM NaCl)	*P. oleracea* L. subsp. *oleracea*	*P. oleracea “Toucan Scarlet Shades”*	*P. grandiflora*	*P. halimoides*
**Chl a**	0	7.82 ± 0.83 bC	3.18 ± 0.21 bA	4.08 ± 0.69 cB	3.25 ± 0.17 aA
	100	9.90 ± 0.67 cC	2.57 ± 0.42 aA	3.27 ± 0.34 bAB	3.76 ± 0.79 bB
	200	7.87 ± 1.13 bC	3.60 ± 0.19 cAB	3.07 ± 0.36 aA	4.28 ± 0.51 cB
	400	5.45 ± 0.29 aB	3.21 ± 0.61 bcA	3.12 ± 0.46 aA	5.57 ± 0.31 dB
**Chl b**	0	5.83 ± 0.97 bC	1.20 ± 0.19 aA	3.13 ± 0.96 bB	1.29 ± 0.24 aA
	100	8.79 ± 1.20 cB	0.95 ± 0.14 aA	1.69 ± 0.41 aA	1.21 ± 0.26 aA
	200	6.09 ± 1.76 bB	1.23 ± 0.08 aA	1.48 ± 0.29 aA	1.23 ± 0.07 aA
	400	4.32 ± 0.42 aB	1.27 ± 0.13 aA	1.41 ± 0.19 aA	1.79 ± 0.14 bA
**Caro**	0	0.49 ± 0.32 aA	0.62 ± 0.04 aB	0.50 ± 0.06 aA	0.66 ± 0.06 aB
	100	0.23 ± 0.29 aA	0.68 ± 0.12 aB	0.52 ± 0.20 aB	0.97 ± 0.25 bC
	200	0.32 ± 0.32 aA	0.91 ± 0.13 bB	0.45 ± 0.15 aA	1.20 ± 0.14 cC
	400	0.42 ± 0.10 aA	0.84 ± 0.23 bB	0.66 ± 0.03 bA	1.78 ± 0.15 dC

**Table 2:** Chloride ions’ concentration in roots and leaves and stems of the four investigated *Portulaca* genotypes undergoing 5 weeks of salt treatments.

Chloride Ions (µmol g^−1^ DW)	Treatment (mM NaCl)	*P. oleracea L.* ssp. *oleracea*	*P. oleracea* “Toucan Scarlet Shades”	*P. grandiflora*	*P. halimoides*
Leaves	0	136.35 ± 25.41aA	244.18 ± 45.78aB	256.34 ± 19.01aB	217.45 ± 46.27aB
	100	1001.88 ± 132.14bC	449.37± 99.91bA	542.94 ± 133.24bA	738.21 ± 20.06bB
	200	1977.47 ± 164.27cC	537.64 ± 108.71bA	691.44 ± 116.61bA	1105.85 ± 50.17cB
	400	2436.03 ± 182.24dC	908.43 ± 88.77cB	618.84 ± 26.94bA	1027.84 ± 248.03cB
Roots	0	357.26 ± 105.75aB	125.47 ± 17.96aA	337.74 ± 64.09aB	N.A.
	100	794.84 ± 181.52bA	710.152 ± 44.68bA	901.01 ± 108.31bA	N.A.
	200	846.95 ± 236.21bA	1444.22 ± 230.83cB	1121.48 ± 211.66bcAB	N.A.
	400	1489.29 ± 119.33cA	2328.77 ± 173.74dB	1336.98 ± 179.31cA	N.A.

Values shown are means ± SE. For each tissue type, different lowercase letters in a column indicate significant differences between treatments; different capital letters in a row denote significant differences among the genotypes undergoing the same treatment.
